# Burkholderia pseudomallei: Challenges for the Clinical Microbiology Laboratory—a Response from the Front Line

**DOI:** 10.1128/JCM.02378-16

**Published:** 2017-02-22

**Authors:** David A. B. Dance, Direk Limmathurotsakul, Bart J. Currie

**Affiliations:** a Lao-Oxford-Mahosot Hospital-Wellcome Trust Research Unit, Microbiology Laboratory, Mahosot Hospital, Vientiane, Lao People's Democratic Republic; b Centre for Tropical Medicine & Global Health, Nuffield Department of Clinical Medicine, University of Oxford, Oxford, United Kingdom; c Faculty of Infectious and Tropical Diseases, London School of Hygiene and Tropical Medicine, London, United Kingdom; d Mahidol-Oxford Research Unit, Faculty of Tropical Medicine, Mahidol University, Bangkok, Thailand; e Menzies School of Health Research, Charles Darwin University and Royal Darwin Hospital, Darwin, Australia; Boston Children's Hospital

**Keywords:** antibiotic, culture, diagnosis, glanders, mallei, melioidosis, prophylaxis, pseudomallei

## LETTER

The minireview by Hemajarata et al. (1) is timely since the global incidence of melioidosis has probably been grossly underestimated (2). However, the review reflects a very U.S. “select agent”-orientated perspective. In some parts of the world, laboratories isolate Burkholderia pseudomallei on an almost daily basis; our own laboratories diagnose more than 600 cases of culture-positive melioidosis each year, giving us a different perspective.

The case described by Hemajarata et al. originated in the Philippines, but they do not mention the fact that only exported cases have been reported in the literature (3–10), implying underdiagnosis of indigenous melioidosis in the Philippines. Unfortunately, even where laboratories exist in areas where melioidosis is endemic, they often misidentify the organism or report it as Pseudomonas sp. (11, 12). We recommend that any oxidase-positive, Gram-negative bacillus that is not obviously Pseudomonas aeruginosa, isolated from any normally sterile clinical specimen, should be tested to exclude B. pseudomallei. In resource-constrained settings, an oxidase-positive Gram-negative rod from a clinical sample that is resistant to gentamicin and polymyxin/colistin but susceptible to co-amoxiclav has a very high probability of being B. pseudomallei, with the exception of isolates from Sarawak, 80% of which are susceptible to gentamicin (as is B. mallei) (13).

In the case reported by Hemajarata et al., postexposure antimicrobial prophylaxis (PEP) was given to nine employees, of whom two developed adverse reactions severe enough to warrant a change of agent. Yet, as the authors note, there have only ever been two well-described cases of laboratory-acquired melioidosis, both of which followed major lapses in laboratory technique (14, 15). More cases of laboratory-acquired glanders have been described (16, 17), implying that B. mallei may present a greater risk to laboratory workers than B. pseudomallei, although many of these occurred at a time when routine biosafety practice was likely to have been less stringent than it is today. Furthermore, the efficacy of PEP in protecting humans from developing melioidosis remains unknown; in animal models, PEP frequently merely delays the onset of disease, rather than preventing it (18). Diagnostic laboratories in areas where melioidosis is endemic handle thousands of isolates of B. pseudomallei at containment levels less stringent than U.S. biosafety level 3, and yet none of us has ever been consulted about a case of confirmed laboratory-acquired infection in over 70 years of combined experience, suggesting that the risk of laboratory-acquired infection with B. pseudomallei is far lower than the risk of infection with some other hazard group 3 agents such as Brucella species or Francisella tularensis. This should be taken into account when conducting risk assessments to inform the need for PEP, the hazards of which are not themselves insignificant; co-trimoxazole, for example, is a well-recognized cause of Stevens-Johnson syndrome (19). We very rarely end up recommending PEP against melioidosis for laboratory workers and suspect that it is likely to have been unnecessary in most, if not all, of the staff in this instance.

There is also confusion about when PEP should be used, which may partly reflect differences between the consensus guidelines of Peacock et al. (20) cited in the review and the earlier *Morbidity and Mortality Weekly Report* guidance that they superseded (21). Hemajarata et al. incorrectly state that PEP is recommended for all low-risk exposures, whereas it is only advised for those who have underlying risk factors that predispose them to melioidosis (20). We believe that a more rational approach to PEP will be achieved if B. pseudomallei is regarded as a naturally occurring, geographically restricted, opportunistic pathogen instead of solely being demonized as a “select agent” (22). Death from melioidosis is very uncommon in healthy people if timely diagnosis, institution of appropriate antibiotics, and state-of-the-art intensive care management are available (23). Nevertheless, there remains a danger that the anxiety generated in this era of “the war on terror”; for example, the “STOP” algorithm for laboratories included in the review by Hemarajata et al. may adversely affect patient diagnosis, management, and outcomes.

We agree that urine and throat swabs should always be cultured for patients with suspected melioidosis. However, nearly one-third of cases will have B. pseudomallei counts of <10^3^ CFU/ml in urine, and it is therefore important to culture the centrifuged deposit, ideally on a selective medium such as Ashdown's agar (not MacConkey agar), for optimal sensitivity (24). Throat swabs also need to be cultured on selective agar such as Ashdown's agar and ideally also pre-enriched in selective broth media (25).

If the results of recent modeling studies are correct, melioidosis is a far bigger killer of humans than diseases that are much better known, such as leptospirosis and dengue (2). As the prevalence of diabetes mellitus increases in areas where melioidosis is endemic and as climate change results in more severe weather events, it is likely to become even more common. Clinical laboratories thus need to be ready to identify B. pseudomallei and think of it as a not uncommon cause of naturally occurring infection in much of the world. We hope that these observations from the “front line” will encourage this.
